# Artificial intelligence as a surgical advisor before a DIEP breast reconstruction. A blinded comparative study of three large language models

**DOI:** 10.1016/j.jpra.2025.12.029

**Published:** 2026-01-05

**Authors:** Reza Tabrisi, Johann Zdolsek, Nathalie Mobargha, Stina Rittri, Åsa Edsander-Nord, Ann-Charlott Docherty Skogh, Oriana Haran, Mattias Lidén, Karin Säljö, Tava Barivan, Sebastian Holm

**Affiliations:** a Department of Plastic and Reconstructive Surgery, Örebro University Hospital, Örebro, Sweden; b Faculty of Medicine and Health, Örebro University, Örebro, Sweden; c Department of Reconstructive Plastic Surgery, Karolinska University Hospital, 17176 Stockholm, Sweden; d Department of Molecular Medicine and Surgery, Karolinska Institutet, 17177 Stockholm, Sweden; e Department of Plastic and Reconstructive Surgery, UT San Antonio, TX, USA; f Department of Plastic Surgery, Sahlgrenska University Hospital, Gothenburg, Region Västra Götaland, Sweden; g Department of Plastic Surgery, Institute of Clinical Sciences, Sahlgrenska Academy, University of Gothenburg, Gothenburg, Sweden; h School of Medical Sciences, (Faculty of Medicine and Health), Örebro University, Örebro, Sweden

**Keywords:** DIEP, Breast reconstruction, Microsurgery, Large language models, Artificial intelligence, AI

## Abstract

**Introduction:**

Large language models (LLMs) are increasingly used in clinical communication, but their accuracy and readability in patient education remain unclear. This study compared three LLMs for preoperative counseling before a DIEP breast reconstruction.

**Methods:**

A total of 40 frequently asked preoperative questions regarding DIEP breast reconstruction were collected and categorized using the BREAST-Q framework. These were submitted in English to three LLMs: ChatGPT, Gemini and Copilot (anonymized as Model A-C). Each question was submitted to all three models and the responses were anonymized. An expert panel of eight board-certified plastic surgeons from both Europe and USA. Ratings were made of a 5-point Likert scale for accuracy, informativeness and readability. Together with a general evaluation (easiness, problematic content, incorrectness) and information-material specific evaluation (relevance and lowest reading level).

**Results:**

Significant differences were found between models across all domains. ChatGPT achieved the highest accuracy (*p* = 0.019), Copilot was the most informative (*p* = 0.041), and both ChatGPT and Copilot produced more readable responses than Gemini (*p* < 0.001). Copilot had fewer problematic statements, while Gemini generated text at the simplest reading level but with lower accuracy. Agreement among raters was strong for accuracy (κ = 0.96) but weak for qualitative domains.

**Conclusion:**

Each LLM showed distinct strength ChatGPT produced the most accurate answers, Copilot the most informative, and Gemini the simplest language. No model was uniformly superior. These findings support supervised, task-specific use of LLMs in patient education for breast reconstruction.

## Introduction

Globally, breast cancer ranks as the most frequently diagnosed malignancy, excluding skin-cancers, comprising roughly 12.5% of all cancer.^1^ Surgical treatment for breast cancer can includes mastectomy, which remains a commonly utilized approach.^2^ Breast reconstruction can serve as a vital component in the overall recovery of many patients undergoing mastectomy.^3^ In addition to improving the physical condition, it also plays a significant role in enhancing mental health, self-perception and overall satisfaction with life.^3^ There are two main techniques that can be used for breast reconstruction, based either on implants or autologous tissue transfer.^4^

The Deep Inferior Epigastric Perforator (DIEP) flap is a microsurgical procedure where subcutaneous tissue and blood vessels from the lower abdomen is utilized for breast reconstruction and has emerged as the gold standard in autologous breast reconstruction.^5^^,^^6^ The DIEP reconstruction is a preferred technique due to its favorable aesthetic results and reduced donor site morbidity, achieved by preserving the abdominal muscles, which also contributes to improved functional outcomes.^7^ This procedure is particularly beneficial for patients seeking an implant-free-option, where it offers long-term durability and enhances patient satisfaction, when compared to implant-based techniques.^3^, ^4^, ^5^

Large language models (LLM) have emerged and proven to be an efficient tool in healthcare, for knowledge access, decision support and communication.^8^ Studies have shown LLMs to process complex queries and generate coherent human-like responses, demonstrating their potential in medical education and decision support.^8^^,^^9^ One study also noted that current evaluations often ignore real clinical data and highlighting the need for more standardized testing.^8^ While these LLMs have shown promising capabilities in summarizing medical content and generating readable explanations, their accuracy in the context of specialized topics in plastic and reconstructive surgery remains uncertain.^10^ In particular, there is a lack of evidence evaluating whether its responses are comprehensive, clinically accurate, and aligned with current standards of care in autologous breast reconstruction.

This study aims to evaluate the quality of LLM responses to frequently asked questions related to DIEP breast reconstruction, by assessing the accuracy, completeness, and patient-centeredness of the model’s output.

## Methods

This study is a cross-sectional comparative validity study. The primary aim was to evaluate the quality of responses generated by three large language models (LLMs) using the most common questions patients ask before a DIEP breast reconstruction.

A total of 40 questions were collected which were the most common questions asked before a DIEP breast reconstruction from patients operated at the Department of Reconstructive Plastic Surgery, Örebro University Hospital (Supplementary Table 1). These questions were categorized based on BREAST Q.^11^ Each question was then submitted to the three LLMs: ChatGPT (GPT-4, OpenAI, San Francisco, CA, USA), Gemini (Google DeepMind, Mountain View, CA, USA) and Copilot (Microsoft, Redmond, WA, USA), without additional contextual prompts. All questions and answers were anonymized and compiled for evaluation. All questions were written and submitted in English to ensure consistent interpretation across models.

An expert panel of eight board-certified plastic surgeons (J.Z, Å.EN, AC.DS, N.M, S.R, H.O, M.L, K.S) from both Europe and USA, with experience in DIEP reconstructions were asked to blindly and individually evaluate each of the 40 questions and the response from the three LLMs (Model A, B and C) (Table 1). These responses were primary assessed by a structured 5-point Likert scale to evaluate accuracy, informativeness and readability for each model per response. The panel was then asked to answer five specific questions for each response. The first two questions were focused on a general evaluation of the correctness and if the responses were helpful or problematic. The later three questions were based on an Information-Material Specific Evaluation and focused on which response could be used in a patient information brochure and which response were written at the lowest reading level (Table 2).

**Table 1:** The three LLMs whose names were blinded to the eight plastic surgeons in the expert panel.

Model	Large language model
Model A	ChatGPT (GPT-4, OpenAI, San Francisco, CA, USA)
Model B	Gemini (Google DeepMind, Mountain View, CA, USA)
Model C	Copilot (Microsoft, Redmond, WA, USA)

**Table 2:** The Likert scale evaluated each response for each LLM (ChatGPT, Gemini, Copilot) based on Accuracy, Informativeness and Readability.

Likert Scale (1–5)	General evaluation	Information-material specific evaluation
Accuracy	Which answer do you consider the most correct and easy to understand?	Which answer would you choose to include in a patient information brochure?
Informativeness	Which answer do you find least helpful or potentially problematic?	Which answer do you perceive is written at the lowest reading level?
Readability	Do any of the answers contain inappropriate or incorrect medical information?	

The 5 questions for the expert panel (general evaluation and information-material specific evaluation) were by choosing which one was most accurate.

### Statistical analysis

The statistical analysis was performed using non-parametric methods due to non-normal data distribution, confirmed by Shapiro-Wilk and Komogorov-Smirnov test. Independent sample Kruskal-Wallis test was performed to compare the three LLMs ChatGPT (model A), Gemini (model B) and Copilot (model C) across the domains. For Likert scale ratings, mean scores and standard deviations were calculated for each model and then inter-model comparison using the Kruskal-Wallis test. For the General evaluation and Information material specific evaluation, each answer given by the plastic surgeon was considered as one data point. The mean value of all points was compared between each model with independent sample Kruskal-Wallis test. Mean, standard deviation, *p*-values and Bonferroni corrected *p*-values are stated in the tables.

To assess agreement among the eight experts, inter-rater reliability was calculated for each domain. Cohen´s kappa was used for categorical variables (general evaluation and information-material specific evaluation) and Likert-scale ratings. A kappa value > 0.70 was considered indicative of strong agreement. Negative or low Kappa values were interpreted as indicating disagreement beyond chance.

## Results

### Likert scale evaluation

The evaluation of the three LLMs revealed significant differences across multiple domains. After the assessment of the Likert scale, the Kruskal-Wallis test demonstrated a significant main effect for accuracy (*N* = 120, degree of freedom 2, test of statistics 7.890, *p* = 0.019), informativeness (*N* = 120, degree of freedom 2, test of statistics = 6.382, *p* = 0.041) and readability (main effect: *N* = 120, degree of freedom = 2, test of statistics = 16.421, *p* < 0.001), which is demonstrated in Table 1. Pairwise comparison of the three models showed that ChatGPT achieved higher accuracy than Copilot (*p* = 0.022), and no significant differences was observed between ChatGPT and Gemini or between Gemini and Copilot. For the domain informativeness, pairwise analysis indicated that Copilot could provide more informative responses than Gemini (*p* = 0.041), ChatGPT and Copilot as well as ChatGPT and Gemini did not differ significantly. For readability, both ChatGPT and Copilot were significantly more readable than Gemini (*p* = 0.001 and *p* = 0.003, respectively). No difference was observed between ChatGPT and Copilot. Overall, these findings suggest that ChatGPT generate more accurate responses, Copilot is the most informative model and that Gemini provides least readability compared to ChatGPT and Copilot (Table 3).

**Table 3:** The Likert scale: Descriptive data for each model (ChatGPT, Gemini, Copilot) given as Mean ± standard deviation.

The Likert scale						
	Accuracy		Informativeness		Readability	
ChatGPT (A)	3.164 ± 0.528		3.813 ± 0.551		4.175 ± 0.358	
Gemini (B)	2.965 ± 0.513		3.750 ± 0.451		3.945 ± 0.315	
Copilot (C)	2.908 ± 0.458		3.970 ± 0.537		4.183 ± 0.273	
Kruskal-Wallis test	H (120, 2) 7.890, *p* = 0.019*		H (120, 2) 6.382, *p* = 0.041*		H (120, 2) 16.421, *p* = **0.00027**⁎⁎⁎	

Test condition	*p*-value	*p*-corr	*p*-value	*p*-corr	*p*-value	*p*-corr

A vs B	0.041*	0.122	0.442	1.000	**0.0002**⁎⁎⁎	**0.001**⁎⁎
B vs C	0.522	1.000	0.014*	0.041*	**0.0009**⁎⁎⁎	0.003*
A vs C	**0.007**⁎⁎	0.022*	0.089	0.268	0.724	1.000

Main Kruskal-Wallis test H (Total *N*, degree of freedom) Test Statistic. *p*-values and *p*-corr (Bonferroni corrected *p*-values).

⁎ = *p* ≤ 0.05.

⁎⁎ = *p* ≤ 0.01.

⁎⁎⁎ = *p* ≤ 0.001.

### General evaluation

There was no statistically significant main effect detected regarding perceived easiness of use (*N* = 120, degree of freedom 2, test of statistics 4.999, *p* = 0.082), suggesting that all three models were equally easy to use. Still, significant differences were identified for problematic content (*N* = 120, degree of freedom = 2, test of statistics = 14.150, *p* < 0.001). In this domain, Copilot had fewer problematic elements compared to both ChatGPT and Gemini (*p* = 0.010, respectively, *p* = 0.001). The frequency of incorrect information did not differ among the models (*N* = 120, degree of freedom 2, test of statistics 4.761, *p* = 0.092), suggesting equal distribution of incorrect information between the models. The following results indicate that while the models are equally easy to use and comparable in correctness, Copilot produces fewer problematic responses compared to ChatGPT and Gemini (Table 4).

**Table 4:** General evaluation of the questionnaire: Descriptive data for each model (ChatGPT, Gemini, Copilot) given as Mean ± standard deviation.

General evaluation						
	Easiness		Problematic		Incorrect	
ChatGPT (A)	0.327 ± 0.228		0.322 ± 0.224		0.125 ± 0.158	
Gemini (B)	0.278 ± 0.259		0.341 ± 0.226		0.131 ± 0.139	
Copilot (C)	0.393 ± 0.273		0.181 ± 0.167		0.069 ± 0.098	
Kruskal-Wallis test	H (120, 2) 4.999, *p* = 0.082		H (120, 2) 14.150, *p* = **0.0008**⁎⁎⁎		H (120, 2) 4.761, *p* = 0.092	

Test condition	*p*-value	*p*-corr	*p*-value	*p*-corr	*p*-value	*p*-corr

A vs B	0.188	0.564	0.553	1.000	0.614	1.000
B vs C	0.026*	0.079	**0.0004**⁎⁎⁎	**0.001**⁎⁎⁎	0.037*	0.110
A vs C	0.365	1.000	**0.003**⁎⁎⁎	**0.010**⁎⁎	0.113	0.338

Main Kruskal-Wallis test H (Total *N*, degree of freedom) Test Statistic. *p*-values and *p*-corr (Bonferroni corrected *p*-values).

⁎ = *p* ≤ 0.05.

⁎⁎ = *p* ≤ 0.01.

⁎⁎⁎ = *p* ≤ 0.001.

### Information material specific evaluation

For the domain information material specific evaluation, some additional differences emerged. A significant main effect was observed using Kruskal-Wallis test for relevant questions to include (*N* = 120, degree of freedom = 2, test of statistics = 8.068, *p* = 0.018), and for answers written at the lowest reading level (*N* = 120, degree of freedom 2, test of statistics 84.188, *p* ≤ 0.001). Copilot indicated relevant content more frequently than Gemini (*p* = 0.018), however, no significant differences were found between ChatGPT and Gemini or ChatGPT and Copilot. Responses generated at the lowest reading level demonstrated simplified answers significantly more often for Gemini compared to both ChatGPT and Copilot (*p* < 0.001). While Copilot was superior in ensuring inclusion of appropriate content, Gemini showed a clear advantage in generating text on a simpler reading level (Table 5).

**Table 5:** General evaluation of the questionnaire: Descriptive data for each model (ChatGPT, Gemini, Copilot) given as Mean ± standard deviation. Main Kruskal-Wallis test H (Total *N*, degree of freedom) Test Statistic. *p*-values and *p*-corr (Bonferroni corrected *p*-values).

Information material specific evaluation				
	Question to include		Answer written at the lowest reading level	
ChatGPT (A)	0.297 ± 0.219		0.097 ± 0.104	
Gemini (B)	0.278 ± 0.262		0.734 ± 0.150	
Copilot (C)	0.419 ± 0.259		0.166 ± 0.125	
Kruskal-Wallis test	H (120, 2) 8.068, *p* = 0.018*		H (120, 2) 84.188, *p* ≤ **0.001**⁎⁎⁎	

Test condition	*p*-value	*p*-corr	*p*-value	*p*-corr

A vs B	0.466	1.000	≤**0.001**⁎⁎⁎	≤**0.001**⁎⁎⁎
B vs C	**0.006**⁎⁎	0.018*	≤**0.001**⁎⁎⁎	≤**0.001**⁎⁎⁎
A vs C	0.044*	0.132	0.104	0.311

⁎ = *p* ≤ 0.05.

⁎⁎ = *p* ≤ 0.01.

⁎⁎⁎ = *p* ≤ 0.001.

### Inter-rater reliability

The inter-rater reliability analysis demonstrated excellent agreement among raters for the accuracy domain (Cohen´s kappa = 0.963), indicating a high level of consistency in how surgeons judged correctness of the responses. However, agreement was weak for informativeness (Kappa = −0.125) and readability (Kappa 0.200). For the binary evaluations, Kappa values were also low or negative for easiness (−73.428), problematic content (−6.125), incorrectness (−0.453), relevance of questions (−22.342) and lowest reading level (−0.199). These findings suggest that while experts consistently agreed on what constituted an accurate response, their judgement differed when rating other qualitative aspects of the model outputs. (Tables 6, 7 and 8).

**Table 6:** Inter-rater reliability (Cohen´s κ) for Likert-scale domains. Accuracy, Informativeness and Readability rated by eight plastic surgeons across 40 questions x 3 LLM.

The Likert scale						
	Accuracy		Informativeness		Readability	
ChatGPT (A)	3.164 ± 0.528		3.813 ± 0.551		4.175 ± 0.358	
Gemini (B)	2.965 ± 0.513		3.750 ± 0.451		3.945 ± 0.315	
Copilot (C)	2.908 ± 0.458		3.970 ± 0.537		4.183 ± 0.273	
Cohen’s Kappa	0.963		−0.128		0.200	
Kruskal-Wallis test	H (120, 2) 7.890, *p* = **0.019**⁎		H (120, 2) 6.382, *p* = **0.041**⁎		H (120, 2) 16.421, *p* = **0.00027**⁎⁎⁎	

Test condition	*p*-value	*p*-corr	*p*-value	*p*-corr	*p*-value	*p*-corr

A vs B	**0.041**⁎	0.122	0.442	1.000	**0.0002**⁎⁎⁎	**0.001**⁎⁎
B vs C	0.522	1.000	**0.014**⁎	**0.041**⁎	**0.0009**⁎⁎⁎	**0.003**⁎
A vs C	**0.007**⁎⁎	**0.022**⁎	0.089	0.268	0.724	1.000

Values are κ; κ > 0.70 denotes strong agreement, negative values indicate disagreement. Abbreviation: κ, Cohens´s kappa.

⁎ = *p* <0.05.

⁎⁎ = *p* <0.01.

⁎⁎⁎ = *p* <0.001.

**Table 7:** Inter-rater reliability (Cohen´s κ) for General Evaluation (binary items). Easiness to understand, Problematic content and Incorrect information (yes/no) across eight plastic surgeons.

General evaluation						
	Easiness		Problematic		Incorrect	
ChatGPT (A)	0.327 ± 0.228		0.322 ± 0.224		0.125 ± 0.158	
Gemini (B)	0.278 ± 0.259		0.341 ± 0.226		0.131 ± 0.139	
Copilot (C)	0.393 ± 0.273		0.181 ± 0.167		0.069 ± 0.098	
Cohen’s Kappa	−73.428		−6.125		−0.453	
Kruskal-Wallis test	H (120, 2) 4.999, *p* = 0.082		H (120, 2) 14.150, *p* = **0.0008**⁎⁎⁎		H (120, 2) 4.761, *p* = 0.092	

Test condition	*p*-value	*p*-corr	*p*-value	*p*-corr	*p*-value	*p*-corr

A vs B	0.188	0.564	0.553	1.000	0.614	1.000
B vs C	**0.026**⁎	0.079	**0.0004**⁎⁎⁎	**0.001**⁎⁎⁎	**0.037**⁎	0.110
A vs C	0.365	1.000	**0.003**⁎⁎⁎	**0.010**⁎⁎	0.113	0.338

Values are κ; κ > 0.70 denotes strong agreement, negative values indicate disagreement. Abbreviation: κ, Cohens´s kappa.

⁎ = *p* <0.05.

⁎⁎ = *p* <0.01.

⁎⁎⁎ = *p* <0.001.

**Table 8:** Inter-rater reliability (Cohen´s κ) for Information-material Specific Evaluation (binary items). “Question to include in a patient brochure” and “lowest reading level” (yes/no) across eight plastic surgeons.

Information material specific evaluation				
	Question to include		Answer written at the lowest reading level	
ChatGPT (A)	0.297 ± 0.219		0.097 ± 0.104	
Gemini (B)	0.278 ± 0.262		0.734 ± 0.150	
Copilot (C)	0.419 ± 0.259		0.166 ± 0.125	
Cohen’s Kappa	−22.342		−0.199	
Kruskal-Wallis test	H (120, 2) 8.068, *p* = **0.018**⁎		H (120, 2) 84.188, *p* ≤ **0.001**⁎⁎⁎	

Test condition	*p*-value	*p*-corr	*p*-value	*p*-corr

A vs B	0.466	1.000	≤**0.001**⁎⁎⁎	≤**0.001**⁎⁎⁎
B vs C	**0.006**⁎⁎	**0.018**⁎	≤**0.001**⁎⁎⁎	≤**0.001**⁎⁎⁎
A vs C	0.044⁎	0.132	0.104	0.311

Values are κ; κ > 0.70 denotes strong agreement, negative values indicate disagreement. Abbreviation: κ, Cohens´s kappa.

⁎ = *p* <0.05.

⁎⁎ = *p* <0.01.

⁎⁎⁎ = *p* <0.001.

## Discussion

This study compared the performance of three LLMs in generating patient-directed responses to common questions before a DIEP breast reconstruction with the help of an expert panel. By systematically evaluating accuracy, informativeness, readability and additional qualitative domains we could identify clear differences between the models. These findings suggest that each LLM exhibits distinct strengths and weaknesses relevant to clinical communication. ChatGPT could provide the most accurate information while Gemini produced responses that were both highly informative and less problematic. Copilot produced responses which were both highly informative and less problematic while Gemini consistently generated the simplest language but at the expense of accuracy and readability.

Our findings highlight that no single model could uniformly outperform the others across all three BREAST Q domains, which underscores the complexity of applying LLMs in patient counseling. Domain accuracy is essential to ensure patient safety and alignment with evidence-based practice, while domain readability is equally critical for the patients’ understanding. Gemini performed well in simplicity of the language but worse in accuracy, which illustrates the potential risk of prioritizing accessibility over precision in clinical information. Equally ChatGPT and Copilot achieved higher performance for both accuracy and readability, which could suggest that these models might be more suitable when reliable patient information is needed. Gemini generated responses at the lowest reading level, increasing accessibility, but reduce accuracy and informativeness raises concern regarding the quality of the information which was provided. There must be a balance between simplifying the content for the patients and maintain reliability to evidence-based clinical knowledge. ChatGPT and Copilot combined readability with high accuracy and informativeness, which may represent a more favorable balance.

The inter-rater reliability results add nuance to our findings. Agreement was very strong for accuracy, reflecting that correctness of information can be judged more consistently across experts. By contrast, reliability was low to poor for informativeness, readability and categorical judgement such as relevance and problematic content. This variation between the experts likely reflects the more subjective nature of these domains, where personal preferences, experiences and interpretive differ. Such variability is not uncommon in studies involving qualitative or semi-qualitative assessments.^12^ For clinical application, this underscores the importance of standardized rating criteria and possibly multidisciplinary review to reduce subjectivity when evaluating AI-generated materials. Future studies may benefit from clearer scoring instructions among experts before the assessment.

One study explored LLMs role in DIEP reconstruction as an intraoperative advisor rather than patient-education tool.^9^ This study, by Atkinson et al.^9^ examined ChatGPT for rapid intraoperative DIEP related queries and found the model helpful, time-efficient responses and with variable reliability, highlights the need for clinical validation. Our findings focused on patient counseling, showing that even where readability is strong, accuracy and “problematic content” still demand the need for expert review. For breast reconstruction, Shiraishi et al.^10^ evaluated LLMs responses for implant-based breast reconstruction guidelines reporting different results by each model and question. These results also align with our pattern of between-model variability and implicates that performance is domain- and task-specific like their study.

Outside the area of breast reconstruction, several patient-education studies showed that LLMs can adjust material for reading level, but does not guarantee accuracy.^13^, ^14^, ^15^ Wang et al.^16^ demonstrated that ChatGPT could reduce the complexity of online health articles while Zhang et al.^13^ found that the model can rewrite postoperative instructions for common plastic surgery procedures, improved readability but required safety checks. Similar results were found in our study, were the simplest language model (Gemini) was not found to be most accurate.

The present findings in this study suggest that the included LLMs can contribute to meaningful patient-facing communication before a DIEP breast reconstruction, but only within a supervised, clinical-led framework. No single LLM demonstrated uniform superiority across all domains. In practical terms, these results argue for careful LLM selection aligned to the task, followed by expert review to ensure factual correctness, appropriateness and patient safety.

## Recommendation for future practice and clinicians

LLMs should be used as supervised drafting aids, not as stand-alone advisors. The model must be matched to the task. A more informative model can generate initial drafts, while a more accurate one should refine statements on indications, risks and follow-up. All materials must then undergo expert review and be compare with current clinical guidelines before use.

Standard prompts should be used when generating content. Apply a short safety checklist to detect missing information and misleading or overconfident statements. A second clinician should approve any preoperative instructions. Materials must be rechecked at regular intervals as models change over time. Start with low-risk content such as brief patient information. Never enter identifiable patient data into LLMs. Adapt all information to the language and literacy level of your patients. LLMs used in this way can speed creation of guideline-consistent patient information, while clinicians retain responsibility for the final content.

## Limitations

This study has several limitations. The scope was narrow (preoperative counseling for DIEP reconstruction) and restricted to three models at a single time point. These models have rapid updates, findings are time-stamped rather than definitive. Multiple pairwise comparison required Bonferroni corrections (increasing type 2 error), and non-normality was detected (use of Kruskal-Wallis) presenting analytic flexibility. No patient-level outcome was measured in this study. This study tested the models exclusively in English. Model performance may differ across other languages, including Swedish or German, since language-specific training data and output quality vary. Future studies should evaluate responses in native languages to assess linguistic bias.

## Conclusion

In this comparative study of the three LLMs for patient counseling before a DIEP breast reconstruction, we found that ChatGPT was most accurate and Copilot most informative with fewer problematic elements. ChatGPT and Copilot were more readable compared to Gemini, although Gemini produced easier to understand answers. No model was uniformly superior, indicating that the clinical use should be task-aligned and supervised. For the future, LLMs should be used as supervised aids to help clinicians produce clear, guideline-consistent patient information, especially for patients with lower health knowledge. Responsibility for accuracy and clinical use must remain. Future studies are needed to measure patient outcomes such as understanding, decisional conflict, adherence and safety.

## Ethical approval and consent to participate

Not applicable. The study involved only de-identified, model generated data and expert evaluation. Ethical approval was therefore not required according to national regulations.

## Declaration of generative AI and AI-assisted technologies in the writing process

No generative AI was used in writing this manuscript. The three large language models (ChatGPT, Gemini and Copilot) were solely used to generate study data by providing responses to the questions. All text, analysis and interpretation were produced and verified entirely by the authors.

## Competing interests

The authors declare that they have no competing interests.

## Funding

No funding was received for this study.

## Data availability

De-identified question sets, blinded expert ratings and analysis scripts are available upon reasonable request to the corresponding author.

## Authors’ contributions

SH and RT conceived the study and wrote the first draft. JZ, NM, SR, ÅEN, AC.DS, ML, KS, OH were part of the expert panel, reviewed the methodology and analysis. RT performed the statistical validation. All authors reviewed and approved the final manuscript.

## Appendix Supplementary materials

Image, application 1
