# Supplementary material for: Artificial intelligence as a surgical advisor before a DIEP breast reconstruction. A blinded comparative study of three large language models
Source: JPRAS Open. 2026 Jan 5;48:669–78. doi: 10.1016/j.jpra.2025.12.029 (PMC12860690; doi:10.1016/j.jpra.2025.12.029)
Supplement: Supplementary file 1 [file mmc1.docx]

**Supplementary material: The 40 questions collected with most common questions asked before a DIEP breast reconstruction.**

**PhysicalWell-Being: Chest and abdomen**

1. **What are the most common and serious risks associated with DIEP flap reconstruction?**
2. Common risks include infection, bleeding, delayed wound healing, fat necrosis (firm or dead fat areas), and fluid buildup (seroma). Serious risks, though rare, include flap loss from blood supply issues, deep vein thrombosis (DVT), or pulmonary embolism.
3. The biggest worry is that the new breast tissue might not get enough blood and could be lost – that's the most serious thing, but it doesn't happen often. More common things include infections in the cuts, fluid build-up in your tummy, or the tummy area not healing well. Sometimes the new breast might not look exactly how you hoped at first.
4. Common risks: Infection, Blood clots, Poor wound healing, Fat necrosis (where some transferred fat doesn’t survive). Serious risks: Loss of the flap due to poor blood supply. Abdominal weakness or hernia, Complications requiring additional surgery. Surgeons monitor your healing closely to catch and manage risks early.

**2. How likely is it that the surgery will not be successful, and what happens then?**

1. Flap failure is relatively rare, occurring in less than 5% of cases. If the flap doesn't survive, surgeons may attempt a revision or consider alternative reconstruction methods.
2. The good news is that this surgery usually works well. But if, for some reason, the new breast tissue doesn't survive, you'd likely need another surgery to remove it. Then, you and your doctors would talk about other ways to rebuild your breast, like using tissue from another part of your body or getting an implant.
3. The success rate of DIEP flap reconstruction is high, but in rare cases, the flap may not survive due to issues with blood supply. If this happens, doctors may attempt another reconstruction or consider other options, like implants or another type of tissue transfer.

**3. How common is it to require reoperation following the initial surgery, and what would that process involve?**

1. Reoperations may be needed in about 10-20% of patients. Reasons include flap revision, scar correction, or fat grafting. These are often minor compared to the original surgery.
2. It's not unusual to need a little "fine-tuning" surgery later on. This could be to make the breasts look more even, fix any small issues with the scars, or rebuild the nipple area.
3. Many patients need minor revision surgeries to refine shape, symmetry, or address small issues like fat necrosis. Major reoperations are less common, but if needed, they might involve adjustments to the flap, scar correction, or additional procedures to improve results.

**4. How do existing conditions such as rheumatoid arthritis, overweight, or underweight affect the risk of complications?**

1. Autoimmune conditions like RA, obesity, and being underweight can increase the risk of delayed healing, infection, and wound complications. Careful preoperative assessment helps minimize risks.
2. These things can make the surgery a bit riskier. Arthritis can slow down healing and increase infection risk. Being overweight can also make healing harder. If you're very thin, you might not have enough tummy tissue for the surgery. Your doctor will check all of this carefully before deciding if it's the right option for you.
3. Rheumatoid arthritis: May affect healing due to immune system changes.
   Overweight**:** Can increase risks of infection, blood clots, or wound healing issues.
   Underweight: May mean less tissue is available, requiring adjustments to the reconstruction method.
   Your surgeon will assess your overall health to minimize risks.

**5. What types of pain can occur (e.g. at the scar, abdomen, or arm), and how long do they usually last?**

1. Pain can occur at the donor site (abdomen), the chest, or around the scars. It is typically most intense in the first 1–2 weeks and gradually subsides over weeks to months. Some patients may experience long-term mild discomfort or sensitivity.
2. You'll likely have pain where you had the mastectomy, on your tummy where they took the tissue, and maybe even a bit in your arm if you had lymph nodes removed. The worst pain is usually in the first few weeks and then gets better over a couple of months. You might have some lingering soreness or nerve sensitivity for longer.
3. Pain is expected but manageable. You may feel:

- Abdominal pain: Due to the removal of tissue, improving within weeks.
- Scar discomfort: Can feel tight or itchy but eases over time.
- Breast soreness: Often fades within a few months.
  Doctors provide pain relief options, including medications, light movement, and support garments.

**6. Is it common to experience numbness in the abdomen or breast, and does sensation usually return?**

1. Yes, numbness is common, especially at the donor site and reconstructed breast. Partial sensation may return over time, but complete return of sensation is not guaranteed.
2. Yes, it's very common to have numbness in your lower tummy where they made the cut. Some feeling usually comes back over many months, but it might not be exactly the same. The new breast will also feel numb at first. You might get some surface feeling back over time, but it probably won't feel like your natural breast.
3. Yes, numbness is normal because nerves are affected during surgery. Sensation may return slowly over months or years, but for some patients, certain areas may always feel slightly numb.

**7. What is the risk of infection, and how can I minimize it at home?**

1. Infection risk is about 5-10%. You can reduce this by following hygiene instructions, keeping incisions clean and dry, taking prescribed antibiotics, and avoiding smoking.
2. The risk of infection is generally low. To help prevent it at home, make sure to wash your hands really well before touching your incisions. Follow your doctor's instructions for changing dressings, keep the area clean and dry, and watch for any signs of redness, swelling, warmth, pain, or pus.
3. Infection risk is low with proper care, but to reduce it:

- Keep the surgical area clean and dry.
- Follow doctor’s wound care instructions.
- Avoid strenuous activity until cleared by your surgeon.
- Watch for signs like redness, swelling, or fever, and contact your doctor if they occur.

8. **How are other parts of the body, such as the back, abdomen, or arms, affected after surgery?**

1. You may feel abdominal tightness, back soreness from walking hunched, or some upper body stiffness. These symptoms usually improve within a few weeks with rest, stretching, and light activity.
2. Your back might feel a bit sore because you'll probably be walking slightly hunched over at first to protect your tummy. Your tummy will definitely feel sore, tight, and maybe a bit weak. Unless you had lymph nodes removed, your arms shouldn't be directly affected by the DIEP flap surgery itself.
3. Different areas of the body may feel tight or sore after surgery:
   Back: May feel stiff due to posture changes while healing.
   Abdomen: Will be tight and sore for a few weeks.
   Arms: Typically unaffected unless lymph nodes are removed.
   Gentle movement and physical therapy can help with discomfort.

**9. What can be done if unexpected complications occur, such as back pain or difficulty standing upright?**

1. Use a walker or supportive device, stretch gently, and do light back exercises under guidance. Pain relief medication and warm compresses may also help during the first few weeks.
2. Tell your doctor right away if you have any unexpected problems. For back pain and trouble standing, they might give you pain medicine and suggest gentle exercises with a physical therapist to help you stand straighter and strengthen your core.
3. Back pain: Often results from adjusting posture; warm compresses and gentle stretching help.
   Difficulty standing upright: Walking slightly bent over for a few days can reduce strain on the abdomen.
   If severe: Your doctor may recommend physical therapy or pain management techniques.

**10. How long do you need to walk bent over after surgery, and how does this affect the back?**

1. Many patients walk slightly hunched for 5–10 days to reduce tension on abdominal incisions. This may lead to temporary back pain, but posture typically returns to normal within two weeks.
2. You'll probably walk a bit bent over for the first few weeks to take pressure off your tummy. This can make your back sore, so it's important to try to straighten up gradually as you feel more comfortable and follow your doctor's advice.
3. Most patients walk slightly bent over for about a week.
   Some back discomfort is common due to changes in posture, but movement improves gradually. Light stretching and support garments help reduce strain.

**11. How long is the expected recovery time before resuming everyday activities?**

1. You can usually resume light activities such as walking or desk work within 4–6 weeks. More strenuous activities, including exercise or lifting, may require 8–12 weeks of recovery.
2. It takes time to recover. You'll likely need a few weeks (maybe 4-6) to rest and get help with daily tasks. It can take several months (3-6 or even longer) to feel fully recovered and get back to all your usual activities.
3. Light activities: Can start within 2-4 weeks.
   Normal daily routine: Usually by 6-8 weeks.
   Strenuous exercise or heavy lifting: Avoid for several months to allow full healing.

**12. How long will I be immobile or severely restricted in movement after surgery?**

1. While rest is crucial, gentle movement is encouraged within 24–48 hours to reduce the risk of complications like blood clots. Activity will be limited for the first two weeks.
2. Right after surgery, you won't be able to move around much because of pain and the need to heal. You'll be encouraged to walk a little to prevent blood clots, but you'll need to avoid heavy lifting and strenuous activity for several weeks (maybe 6-8 or longer).
3. The first few days require rest, but movement is encouraged. Walking helps prevent complications like blood clots, but heavy lifting and intense movement should be avoided for 6+ weeks.

**13. How does the surgery impact sleep and mobility immediately afterward?**

1. You may struggle to find a comfortable sleeping position. Many patients sleep in a recliner or with pillows propping them up for the first 1–2 weeks. Movement will feel stiff and slow initially.
2. Sleeping might be hard at first because of discomfort. You'll probably need to sleep on your back or propped up with pillows. Moving around will also be difficult, and you'll need help getting in and out of bed and walking.
3. Sleeping in a slightly elevated position helps reduce swelling. Mobility is restricted for a few days, but light walking is encouraged.
   Getting in and out of bed may feel difficult at first, but support devices and slow movements help.

**14. How should the operated area be cared for at home to ensure proper healing?**

1. Follow your care team’s instructions carefully. Clean wounds with sterile supplies, monitor for signs of infection, change dressings as advised, and wear abdominal binders if recommended.
2. Keep the cuts clean and dry, change dressings as your doctor tells you, watch for signs of infection, avoid straining your incisions, take your pain medicine, and wear any supportive garments your doctor recommends.
3. Keep incisions clean and dry. Avoid heavy lifting or stretching early on. Wear compression garments if recommended. Follow up with your surgeon for check-ups.

**15. How much help will I need at home during the first few weeks after surgery?**

1. Plan for help with meals, personal care, and chores for the first 1–2 weeks. Most people regain basic independence by week 3 or 4, though lifting and driving may still be restricted.
2. You'll need quite a bit of help at home for the first couple of weeks with things like showering, dressing, making food, and getting around. Having someone there to support you is really important.
3. Assistance is needed for daily activities like cooking, cleaning, and dressing for at least the first 1-2 weeks. If you live alone, arranging help is important.

**16. How long will it take for the pain to ease, and how can pain be managed?**

1. Pain typically lessens within the first two to three weeks. It’s managed with prescription or over-the-counter painkillers, ice packs, rest, and gradual movement.
2. The worst pain usually lasts for the first few weeks and then slowly gets better over a couple of months. Your doctor will give you pain medicine to take. You can also use ice packs to help with swelling and pain.
3. Pain gradually improves within weeks. Management includes: Prescribed medications (painkillers, anti-inflammatories). Support garments to ease discomfort. Gentle movement to prevent stiffness. Ice packs (if approved by your doctor) for swelling.

**17. What are the risks of complications during the healing process, and how are they handled?**

1. Risks include infection, seroma, hematoma, or wound dehiscence. These are usually managed with antibiotics, drainage, wound dressings, or minor surgical revision if needed.
2. Things like infection, fluid build-up, blood clots, or the wound opening up can happen during healing. Your doctor will watch for these and treat them if they occur, possibly with antibiotics, draining fluid, or more wound care.
3. While most patients heal well, complications can arise. The most common issues include: Infection, which may require antibiotics. Poor wound healing, leading to delayed recovery or the need for revision surgery. Seromas, which are fluid collections that may need draining. Fat necrosis, where some of the transferred fat does not survive, sometimes requiring adjustments later. Doctors monitor healing closely and recommend treatments as needed to ensure recovery goes smoothly.

**18. What does the recovery process look like for patients who have difficulty healing?**

1. Slow healing may require extended follow-up, wound care treatments, or physical therapy. Maintaining a healthy diet, hydration, and avoiding smoking can promote better healing.
2. If you have trouble healing, it might take longer to recover, and you might have more pain or a higher risk of infection. Your doctors will try to figure out why you're not healing well and give you extra care, like special wound treatments or nutritional support.
3. Some patients take longer to heal due to medical conditions, smoking history, or other factors. If healing is slow, doctors may adjust medications to support better wound healing, recommend physical therapy to help regain mobility, perform minor procedures to address wound complications, provide additional monitoring through follow-up visits.
   Patience is key, and your healthcare team will support you through the process.

**19. Is there a risk of swelling in the mons pubis (the area above the pubic bone) after surgery?**

1. Yes, temporary swelling in the mons pubis area is common after abdominal surgery. It usually resolves in a few weeks. Persistent swelling may require medical evaluation.
2. Yes, it's possible to have some swelling in that area after surgery due to fluid shifts. It usually goes away on its own over time.
3. Yes, swelling in the mons pubis is common due to fluid buildup after surgery. It usually improves over several weeks as your body heals.
   To reduce swelling:

- Wear compression garments as recommended.
- Move regularly to improve circulation.
- Elevate the lower body when resting to help drainage.
  If swelling persists or worsens, your surgeon can evaluate and provide guidance.

**20. What exercises or measures can help strengthen the abdomen again after surgery?**

1. After 6–8 weeks, light core exercises like pelvic tilts, leg slides, or guided physical therapy routines can help. Always get your surgeon’s clearance before starting exercise.
2. Gentle walking is good to start. Later, a physical therapist will give you exercises to slowly strengthen your tummy muscles. Don't do any hard tummy exercises until your doctor says it's okay.
3. Once fully healed, gentle core-strengthening exercises help rebuild abdominal strength. These may include:

- Walking regularly to regain mobility.
- Pelvic tilts to strengthen lower abdominal muscles.
- Gentle stretching to improve flexibility.
- Low-impact activities like yoga, under medical supervision.
  Avoid strenuous abdominal exercises until cleared by your surgeon.

**Psychosocial Well-Being**

**21. How can patients mentally prepare for the extensive care and recovery process?**

1. Talk to others who’ve had the surgery, make a recovery plan, understand the expected timeline, and create a strong support network. Journaling or counseling may also help.
2. Try to learn as much as you can about the surgery and recovery. Have a good support system in place. Be patient with yourself – it takes time to heal. Maybe talk to other people who've had this surgery or consider talking to a therapist if you're feeling overwhelmed.
3. Preparing mentally is just as important as physical recovery.

- Set realistic expectations—recovery takes time, and small progress is meaningful.
- Seek support through friends, family, or patient groups.
- Talk to your healthcare team about concerns and fears.
- Consider relaxation techniques like meditation or deep breathing to manage stress.
  Emotional preparation can make the recovery journey easier.

**22. How can I get better information and support about feeling vulnerable or dependent after surgery?**

1. Reach out to your care team, social workers, or hospital support services. Many cancer centers offer peer support or buddy programs to help you navigate the emotional journey.
2. Talk to your doctors and nurses about how you're feeling. Lean on your family and friends. Consider joining a support group where you can connect with others who understand what you're going through. A therapist or counselor can also provide a lot of help.
3. Many patients feel vulnerable or dependent in the early recovery phase. Support is available through: Patient forums where others share their experiences. Counseling services if emotional challenges arise. Nurses or care coordinators, who provide recovery guidance.
   Acknowledging these feelings and reaching out for help can make a big difference.

**23. What kind of emotional support is available for patients feeling isolated during recovery?**

1. Join online or in-person support groups, talk to a therapist, or use hospital-provided counseling services. Staying connected can help reduce feelings of loneliness.
2. Support groups (online or in person) can help you connect with others. Online forums and communities can also provide a sense of belonging. Don't hesitate to talk to your healthcare team or a therapist about your feelings.
3. Recovery can sometimes feel isolating, but there are ways to stay connected:

- Online support groups where patients share experiences.
- Counseling or therapy to process emotions.
- Friends or family visits, even if virtual, to maintain connection.
  Seeking social interaction, even in small ways, can help ease feelings of isolation.

**24. How common is it to feel concerned about the symmetry or appearance of the breasts after surgery?**

1. It’s very common. Speak with your surgeon about your concerns. Secondary surgeries or fat grafting can often address cosmetic issues.
2. It's very common to have concerns about how your breasts look after surgery, especially if they don't look perfectly even right away.
3. It is very common for patients to have concerns about breast symmetry and appearance. Surgeons aim for the best balance possible, but minor differences can occur. If symmetry is a concern after healing, minor revision procedures can help.

**Satisfaction With Breasts / Aesthetic Outcomes**

**25. How symmetrical are the breasts typically after DIEP flap reconstruction?**

1. Surgeons aim for the best possible symmetry, but perfect results are rare. Any noticeable asymmetry can often be corrected in a follow-up procedure.
2. While surgeons try their best to make them as even as possible, it can be tricky to get them perfectly symmetrical. You might need another small surgery later to improve the appearance.
3. Surgeons aim to create well-balanced breasts, but no reconstruction is perfectly symmetrical. Factors like swelling, healing variations, and tissue adjustment influence final results. Over time, adjustments or minor revisions can be made if needed.

**26. What is the risk that one breast will end up larger than the other after surgery?**

1. Minor size differences are common and often improve with fat grafting or revision surgery. Larger discrepancies may be corrected by adjusting the opposite
2. There's a chance they might not be exactly the same size. Swelling and how your body heals can affect the final size. If the difference is significant, another surgery can sometimes help.
3. It is possible for one breast to be slightly larger due to how the transferred tissue settles. If there is a significant difference, adjustments like fat grafting or reduction procedures may be performed later.

**27. What do typical scars look like on the abdomen and breast after the procedure?**

1. Expect a low horizontal scar across your lower abdomen and scars on or around the breast. Scars usually fade over 6–12 months but remain visible to some degree.
2. On your tummy, you'll have a scar across your lower abdomen, kind of like a tummy tuck scar, and a scar around your new belly button. On your breast, you'll have scars around the edges of the new breast, and maybe some smaller scars from the drains. Scars usually fade over time but will still be visible.
3. Scars are permanent but fade over time.

- Abdomen: Horizontal scar along the lower belly.
- Breast: Scars vary based on technique; they often follow natural contours.
  Surgeons aim to position scars discreetly, and some patients pursue scar treatments for further improvement.

**28. If I am slim and do not have enough tissue on the abdomen, what happens? Will tissue need to be taken from another area, or will the other breast need to be reduced?**

1. If abdominal tissue is insufficient, alternative donor sites like the thighs or buttocks can be used. Breast reduction on the opposite side is sometimes considered to match volume.
2. If you don't have enough tummy tissue, your doctor might suggest taking tissue from another part of your body, like your thighs or buttocks. Another option could be to reduce the size of your other breast to match the reconstructed one, or to consider an implant.
3. If you do not have enough abdominal tissue, surgeons may use other donor areas, such as the thigh or buttocks, or consider reducing the size of the natural breast for better balance.
   Your surgeon will explore the best approach for your body type.

**29. How similar will the reconstructed breast be to a natural breast in shape, feel, and appearance?**

1. The reconstructed breast will be soft and warm but won’t feel exactly like your natural breast, especially due to reduced sensation. Visually, it may look quite similar under clothing.
2. A DIEP flap can feel and look quite natural because it's made from your own tissue. It will move and change more like a natural breast over time. However, it won't have the same sensation as your original breast.
3. The reconstructed breast looks and feels natural, but differences exist.

- Shape: Similar, but may be firmer than a natural breast.
- Feel: Softer than an implant but without full sensation.
- Appearance: Close, but aging may differ slightly over time.
  Most patients are satisfied with the results, though adjustments may be made for fine-tuning.

**30. Will I receive a new nipple immediately, or is that done at a later stage?**

1. Usually not. Nipple reconstruction is typically done in a separate outpatient procedure once healing is complete, or via tattooing.
2. Usually, the nipple and the darker skin around it (areola) are reconstructed in a separate surgery, a few months after the main breast reconstruction. This allows the new breast to settle first.
3. Nipple reconstruction is not done immediately. It is performed in a later procedure, once the breast shape has fully healed.

**31. How does the natural breast and the reconstructed breast compare in appearance, especially if the natural breast is larger?**

1. When one natural breast remains, especially if it’s larger, it may appear different from the reconstructed breast in terms of shape, fullness, and position. The reconstructed breast, although made from your own tissue, may be firmer or have less droop compared to the natural side. In such cases, surgeons might recommend a breast reduction or lift on the natural breast for better symmetry, either during the initial surgery or as a later procedure. Under clothing, many patients report the appearance is quite natural and balanced.
2. It can be challenging to make the new breast exactly match a larger natural breast. Your surgeon will do their best during the surgery, but you might need a later surgery on either breast to get better symmetry.
3. If the natural breast is larger, adjustments like a breast reduction or fat grafting may be suggested to improve balance. Surgeons aim for as much symmetry as possible.

**32. How will the abdomen look after the surgery if the belly button needs to be moved?**

1. Your abdomen will have a low, hip-to-hip scar similar to a tummy tuck and a scar around the relocated belly button. Most patients are satisfied with the cosmetic result.
2. Your tummy will look flatter, similar to after a tummy tuck. There will be a scar where your old belly button was and a new scar around your relocated belly button.
3. The belly button may be repositioned during surgery. It will appear natural, but a small scar may be present.

**33. How does the reconstructed breast change over time compared to a natural breast with aging?**

1. DIEP flap breasts tend to hold their shape well over time, but natural aging or weight fluctuations may lead to slight changes in appearance.
2. Because it's made of your own tissue, the reconstructed breast will likely change over time in a similar way to your natural breast, for example, with weight changes or aging.
3. The reconstructed breast ages differently because it lacks glandular tissue. It may not sag as much as a natural breast but remains stable in shape.

**34. Is swelling of the mons pubis (area above the pubic bone) common after surgery?**

1. Yes, temporary swelling in the mons pubis area is common after abdominal surgery. It usually resolves in a few weeks. Persistent swelling may require medical evaluation.
2. Yes, it's fairly common to have some swelling in that area after surgery, and it usually gets better on its own.
3. Yes, temporary swelling occurs but improves in a few weeks.

**Satisfaction With Care (Practical Care Questions)**

**35. How much help is needed in daily life immediately after surgery?**

1. You’ll need full-time assistance for the first week with basic tasks such as getting in and out of bed, bathroom use, dressing, meal prep, and medication management.
2. You'll need a lot of help with everyday tasks right after surgery, like getting out of bed, showering, dressing, and preparing food.
3. Help is needed for at least 2 weeks with household tasks, mobility, and self-care.

**36. What happens immediately after surgery - when do you go to the hospital ward, and how long are you monitored?**

1. You’ll be closely monitored in a recovery area, where nurses will check your flap circulation frequently. Once stable, you’ll be moved to a hospital room for several days for ongoing care and observation.
2. After surgery, you'll wake up in a recovery room where they'll watch you closely. Once you're stable, you'll go to a regular hospital room. You'll likely stay in the hospital for a few days so they can monitor the blood flow to the new breast, manage your pain, and make sure you're healing okay.
3. Patients go to recovery immediately after surgery and stay in the hospital for several days for monitoring.

**37. How does the surgery affect sleep and the ability to move immediately afterward?**

1. Expect to feel stiff and uncomfortable at first. Sleep can be challenging due to incision sites and limited mobility, but using pillows or sleeping in a recliner may help. Movement improves as swelling and soreness decrease.
2. As mentioned before, sleep will probably be uncomfortable, and moving around will be difficult at first.
3. Sleeping may be difficult at first. Movement improves gradually.

**38. What is the process with drainage tubes after surgery, and can it be painful or traumatic to remove them?**

1. After DIEP surgery, small drainage tubes are placed to remove extra fluid. They stay in for about 1–2 weeks. Taking them out is usually quick and not very painful—just a strange feeling or a little pinch. Most people say it’s over fast and not a big deal.
2. You'll have tubes placed in the surgery areas to drain extra fluid. They're usually connected to little containers. The nurses will show you how to empty them. Taking them out usually isn't very painful, just a bit of a pulling sensation.
3. Drainage tubes help remove excess fluid. Removal is uncomfortable but tolerable.

**39. How much sensation remains in the new breast, and is there a chance for sensation to return?**

1. Most sensation is lost immediately after surgery. While some patients regain partial feeling over time, especially with nerve reconnection techniques, full sensation is rarely restored.
2. Right after surgery, you'll have very little or no feeling in the new breast. Over time, you might get some surface sensation back as nerves grow, but it probably won't feel like your natural breast.
3. Sensation varies, and some feeling may return over time.

**40. How long does the surgery itself usually take?**

1. You’ll be closely monitored in a recovery area, where nurses will check your flap circulation frequently. Once stable, you’ll be moved to a hospital room for several days for ongoing care and observation.
2. DIEP flap surgery is a big surgery and can take several hours, usually between 4 and 8 hours, depending on whether you're having one or both breasts reconstructed.
3. DIEP flap surgery typically lasts 6-8 hours.
